# Inhibition Effect of Solid Products and DC Breakdown Characteristics of the HFO1234Ze(E)–N_2_–O_2_ Ternary Gas Mixture

**DOI:** 10.1021/acsomega.1c03020

**Published:** 2021-08-31

**Authors:** Heng Liu, Qingmin Li, Jingrui Wang, Yuheng Jiang, A. Manu Haddad

**Affiliations:** † State Key Laboratory of Alternate Electrical Power System with Renewable Energy Sources, North China Electric Power University, Beijing 102206, China; ‡ School of Electrical and Electronic Engineering, North China Electric Power University, Beijing 102206, China; § Advanced High Voltage Engineering Research Centre, Cardiff University, Cardiff, Wales CF24 3AA, United Kingdom

## Abstract

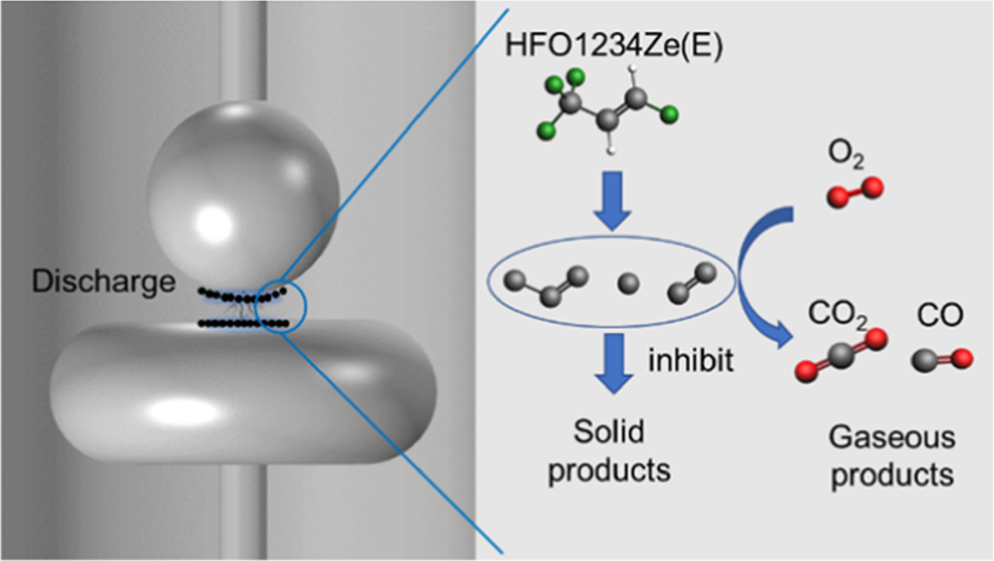

HFO1234ze(E) is an environmentally friendly SF_6_ substitute gas with prominent application potential. To suppress the generation of the HFO1234ze(E) solid decomposition products, which may cause great hazards to the gas–solid insulation strength, a gas mixing scheme screening method based on the reactive force field (ReaxFF) molecular dynamics (MD) simulation was innovatively proposed. The simulation results show that the inhibitory effect of O_2_ on the formation of HFO1234ze(E) solid products is better than those of CO_2_ and CF_4_. Further study shows that when O_2_ accounts for 3.33% of the gas mixture, the solid precipitate content is reduced by 48%. The experimental study shows that an O_2_ content of 3.33% can inhibit the generation of solid products by more than 50%. Besides, compared with HFO1234ze(E)-N_2_, the DC breakdown voltage of HFO1234ze(E)-N_2_-O_2_ is slightly increased, and the breakdown voltage dispersion degree and continuous breakdown voltage drop rate are decreased. This work gives a feasible solution for the suppression of HFO1234ze(E) solid decomposition products and provides an efficient method for solving similar problems of environmentally friendly insulating gas in C/F/O/N systems.

## 1 Introduction

Due to its high dielectric strength and excellent arc extinction performance, sulfur hexafluoride (SF_6_) has been widely used in power transmission and distribution equipment, such as gas-insulated switchgear (GIS) and gas-insulated transmission line (GIL).^1,2^ However, the global warming potential (GWP) of SF_6_ is 23 900 times that of CO_2_ and its atmospheric lifetime is as long as 3200 years.^3^ SF_6_ that escapes into the atmosphere will exacerbate the greenhouse effect in a quite long period of time. For environmental protection and sustainable development considerations,^4,5^ it has become an urgent need to find an environmentally friendly insulating gas to replace SF_6_.

In recent years, researchers have made some progress in searching for potential SF_6_ substitute gases. Some alternative candidates such as c-C_4_F_8_,^6^ CF_3_I,^7^ C_5_F_10_O, and C_4_F_7_N^8^ were identified because of their strong insulation properties (equivalent to or stronger than SF_6_). However, the shortcomings of the aforementioned candidate gases in terms of the greenhouse effect (c-C_4_F_8_), material compatibility (CF_3_I), and toxicity (C_5_F_10_O and C_4_F_7_N) limit their wide application to some extent. Recently, HFO1234Ze(E) (referred to as hydrofluoroolefins (HFO) when it does not cause ambiguity), one of the hydrofluoroolefins family gases, shows favorable properties in replacing SF_6_ in medium voltage systems.^9,10^ The GWP of this gas is very low (GWP = 6) and its ozone depletion potential is zero.^10^ The experimental results indicate that the breakdown voltage of HFO is 74–94% of SF_6_ under identical experimental conditions. In addition, as a refrigerant with mature applications, it is found to be nontoxic, noncarcinogenic, non-reprotoxic, and nonmutagenic.^11^ Since the boiling point of pure HFO gas is −19 °C,^12^ a buffer gas is needed to solve the problem of low-temperature liquefaction. The experimental study shows that the AC breakdown voltage of the HFO–N_2_ mixture under a slightly nonuniform electric field is close to that of the SF_6_–N_2_ mixture.^13^

It should be noted that in experimental studies, black solid precipitates are observed to scatter near the discharge location after the discharge failure. Although some researchers have pointed out that a small amount of solid precipitates covering the electrode surface has no significant effect on the breakdown strength of the insulating gas,^14^ if the discharge location is near the insulator, the solid precipitates are easily dispersed on the surface of the insulating material, which may cause great hazards to the surface insulation strength. Therefore, exploring the method for suppressing the HFO solid precipitates is one of the key issues that need to be resolved before the reliable replacement of SF_6_ by HFO gas. The existing literature studies have shown that the introduction of a buffer gas to adjust the gas decomposition characteristics is a potential solution to suppress the solid products. Considering that the efficiency of finding suitable buffer gases and optimal mixing schemes that can inhibit the HFO solid decomposition products through experiments is very low, it is imperative to explore a more effective research method.

After nearly 20 years of development, the molecular dynamics (MD) simulation based on the reactive force field (ReaxFF) has been very mature.^15^ Compared with the classical molecular force fields, ReaxFF introduces the concept of bond order (BO) to determine the covalent interaction between the atoms. By using BO, it is unnecessary to fix the connectivity of atoms so that the chemical bonds between atoms can be freely broken or formed.

In recent years, ReaxFF MD simulation has been widely used in the research of insulation gas decomposition mechanisms. Li et al. studied the decomposition characteristics of the environmentally friendly insulating gas C_5_F_10_O and analyzed the influence of the buffer gas (air) on the generation of C_5_F_10_O decomposition products.^16^ Zhang et al. explored the decomposition mechanism of the C_4_F_7_N and the C_4_F_7_N/CO_2_ gas mixture.^17,18^ Liu et al. studied the effect of O_2_ on the decomposition products of SF_6_ and explained the generation mechanism of its oxygen-containing decomposition products.^19^ Lin et al. studied the decomposition characteristics of the HFO/N_2_ mixture.^13^ In addition, ReaxFF has also been used to explore the decomposition mechanism of the hydrofluoroolefins family working fluids. Huo et al. studied the oxidation decomposition mechanism of HFO1336mzz(Z).^20^ Pu et al. studied the pyrolysis mechanism of HFO-1234yf (an isomer of HFO1234Ze(E)).^21^ On the basis of the above-mentioned research work, it is a feasible research approach to use ReaxFF MD simulation to guide the screening of buffer gases that could inhibit the precipitation of HFO solid decomposition products from the perspective of the microreaction mechanism.

In this paper, simulation and experimental research were carried out on the suppression methods of HFO solid decomposition products for the first time. This research work mainly focuses on screening suitable buffer gases to regulate the microscopic decomposition process of HFO gas, and finally, achieve the suppression of solid precipitates. To achieve this research goal, we innovatively proposed a research method for evaluating the inhibition effect of the HFO solid decomposition products by buffer gases based on ReaxFF MD simulation, which greatly improved the efficiency of the buffer gas screening process. Through ReaxFF simulation, the inhibitory effects of several candidate buffer gases on HFO solid decomposition products were simulated, and the influence mechanism of various factors on the inhibitory effects of solid products was analyzed. Furthermore, experiments were carried out to study the DC breakdown characteristics of the HFO ternary gas mixture and the suppression effect of solid products. Based on the above research, this work provides a gas mixing scheme that can effectively inhibit the HFO solid decomposition products, which could provide technical support for promoting HFO to replace SF_6_ in the medium voltage equipment.

## 2 Results and Discussion

### 2.1 Selection of a Buffer Gas to Inhibit HFO Solid Decomposition Products

#### 2.1.1 Inhibitory Effect of Different Buffer Gases on HFO Solid Precipitates

According to the existing research results,^22^ the C–F, C–H, and C–C bonds in HFO molecules may break and generate many C, C_2_, or C_3_ particles under the action of discharge and high temperatures. When these particles recombine with each other to form clusters with a large number of carbon atoms during the cooling process, they will precipitate out of the gas reaction system and become solid products. From the above analysis, it can be seen that the fundamental way to inhibit the formation of HFO solid precipitates is to reduce the probability of the formation of carbon clusters. It can be considered from two aspects: (1) inhibit the generation of C*_x_* particles and (2) promote the conversion of C*_x_* particles into other gaseous products. Based on the research experience from the literature, three potential buffer gases, CO_2_, O_2_, and CF_4_, are selected for further research in this article. Since HFO1234Ze(E) is not flammable and has no flash point according to standards, the addition of O_2_ does not constitute a safety risk.

**Figure 1:**
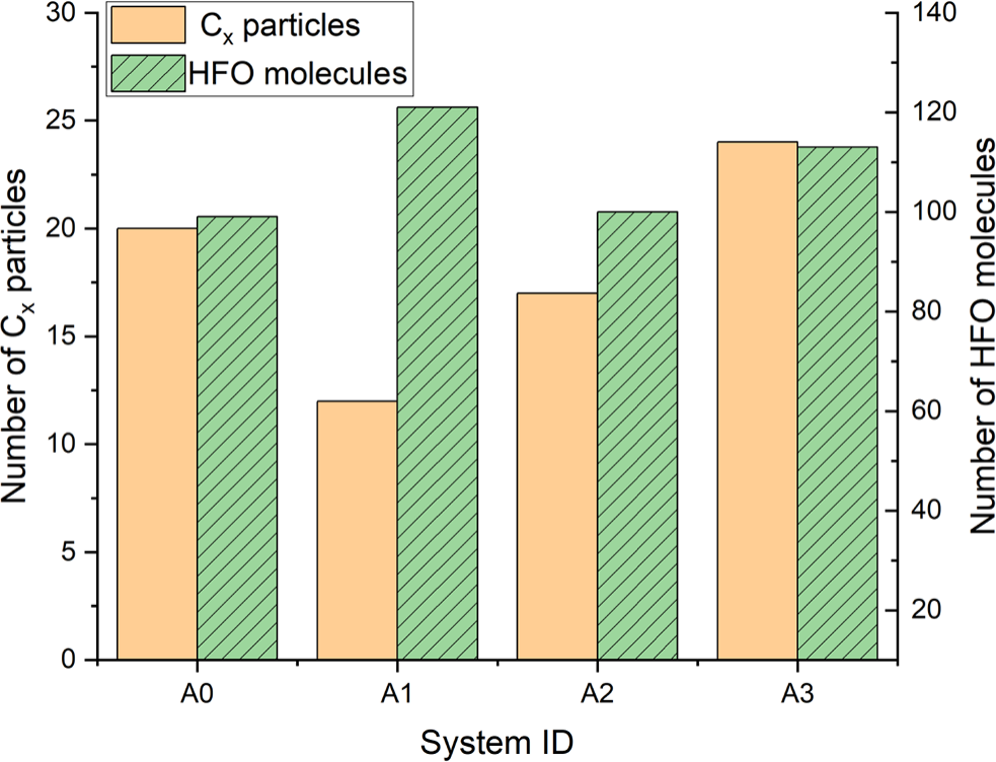
ReaxFF simulation results for the HFO mixture with different buffer gases.

After performing 1000 ps simulation according to the settings of Section 4.1, the number of remaining HFO molecules and the number of C*_x_* particles in the A0–A3 system are shown in Figure 1 (to reduce the influence of fluctuations (±2) in the number of C*_x_* particles, the average value of 900–1000 ps is taken for analysis).

It can be seen from Figure 1 that the number of C*_x_* particles generated in the A1 and A2 systems is less than that in the A0 system, while the A3 system generates the most C*_x_* particles. On the other hand, the decomposition ratio of HFO in the A1 and A3 systems is lower than that in the A0 system. Meanwhile, the decomposition degree of HFO in the A2 system is basically the same as that in the A0 system. However, the amount of C*_x_* particles generated in the system is directly related to the amount of solid precipitation,^23^ and the degree of HFO decomposition, at the same time, is also closely related to the gas insulation performance. After comprehensively considering these two factors, it can be considered that the A1 system (HFO–N_2_–O_2_ gas mixture) has a better solid precipitate suppression effect and application potential.

In addition to the three candidate buffer gases (O_2_, CO_2_, CF_4_) considered in this study, there are many other potential buffer gases that may achieve the goal of suppressing the HFO solid products. Therefore, a detailed analysis of the reasons for obtaining the above simulation results will help to understand the microscopic decomposition mechanism of the HFO gas mixture system under the action of different buffer gases, which can provide more helpful information for the screening of other possible buffer gases that could inhibit the precipitation of HFO solid products.

#### 2.1.2 Influence Mechanism of Buffer Gases on the HFO Solid Decomposition Products

As mentioned above, reducing the decomposition of HFO and accelerating the consumption of C*_x_* particles are the main ideas for suppressing the solid decomposition products of HFO. To analyze the impact mechanism of the buffer gas on the degree of decomposition of HFO, we first obtained the main decomposition path of HFO by tracking the motion trajectory of molecules (atoms) in the simulation process, as shown in Table 1.

**Table 1:** Typical Decomposition Paths of HFO and its Reaction Rate Constants^a^

ID	reaction path	reaction rate constant
(1)	C_3_H_2_F_4_ → C_3_HF_4_ + H	6.51 × 10^–8^
(2)	C_3_H_2_F_4_ → C_3_H_2_F_3_ + F	3.91 × 10^–8^
(3)	C_3_H_2_F_4_ → CF_3_ + H + C_2_HF	2.58 × 10^–8^
(4)	C_3_H_2_F_4_ + H → C_3_H_3_F_4_	1.22 × 10^–9^
(5)	C_3_H_2_F_4_ + F → C_3_H_2_F_5_	7.78 × 10^–10^

a *Note*: the molecular formula of HFO1234Ze(E) is C_3_H_2_F_4_.

The reaction paths (1), (2), and (3) given in Table 1 are all simple bond-breaking reactions, so their reaction rates mainly depend on the physicochemical properties of the HFO molecule itself and the external factors such as temperature. These reaction paths will play a leading role in the initial decomposition of HFO. As the reaction progresses, the concentrations of H, F, and CF_3_ radicals in the system will affect the reverse reaction rates of the reaction paths (1), (2), and (3) to a certain extent. For reaction paths (4) and (5), the reaction rates are mainly affected by the H atoms and F atoms generated by the decomposition of HFO in the system. Comparing the reaction rate constants in Table 1, it can be found that free H atoms have a greater influence on the decomposition of HFO than free F atoms in the subsequent reaction process.

**Figure 2:**
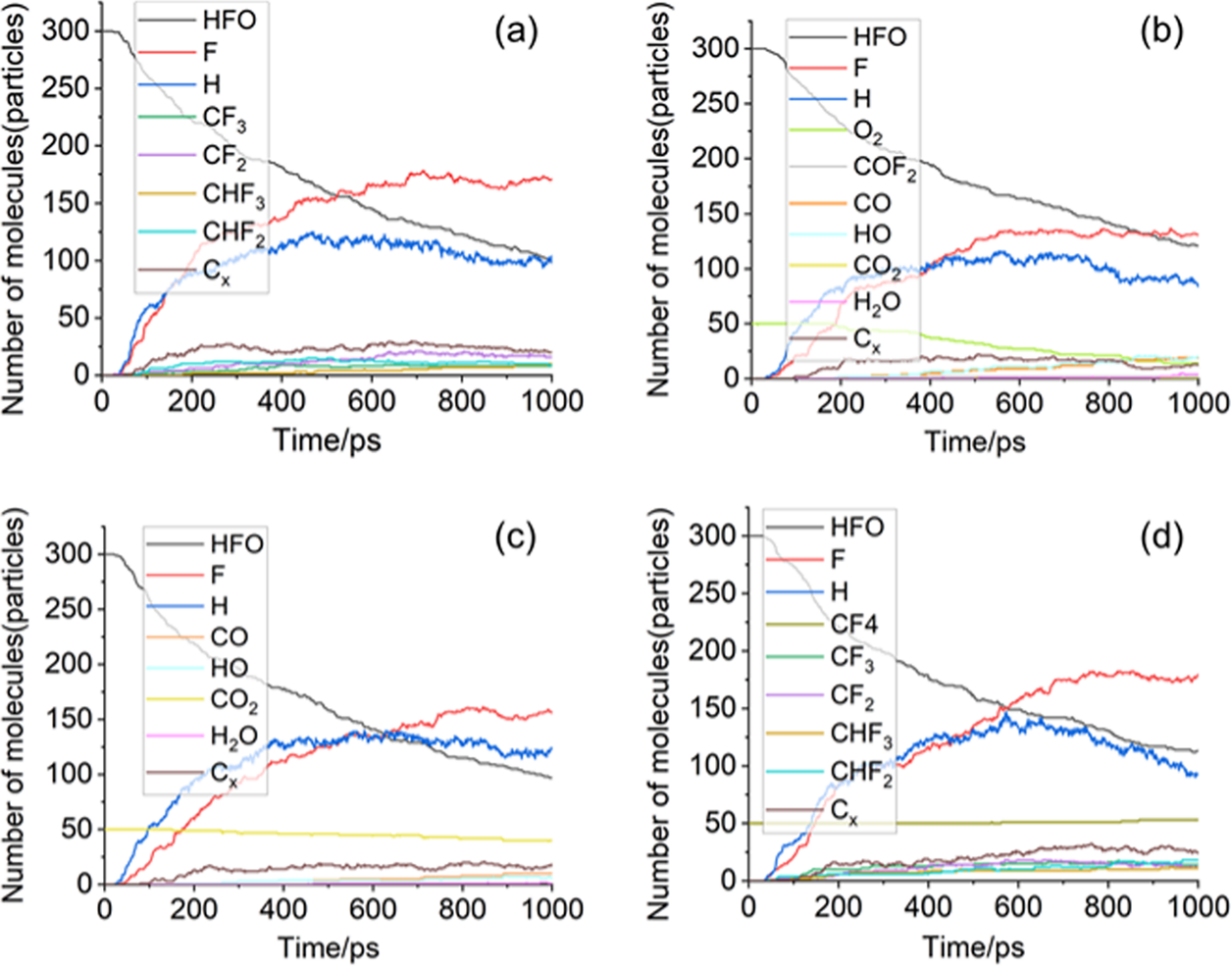
Change curves of reactants and the main decomposition products in the (a) A0 system, (b) A1 system, (c) A2 system, and (d) A3 system.

**Figure 3:**
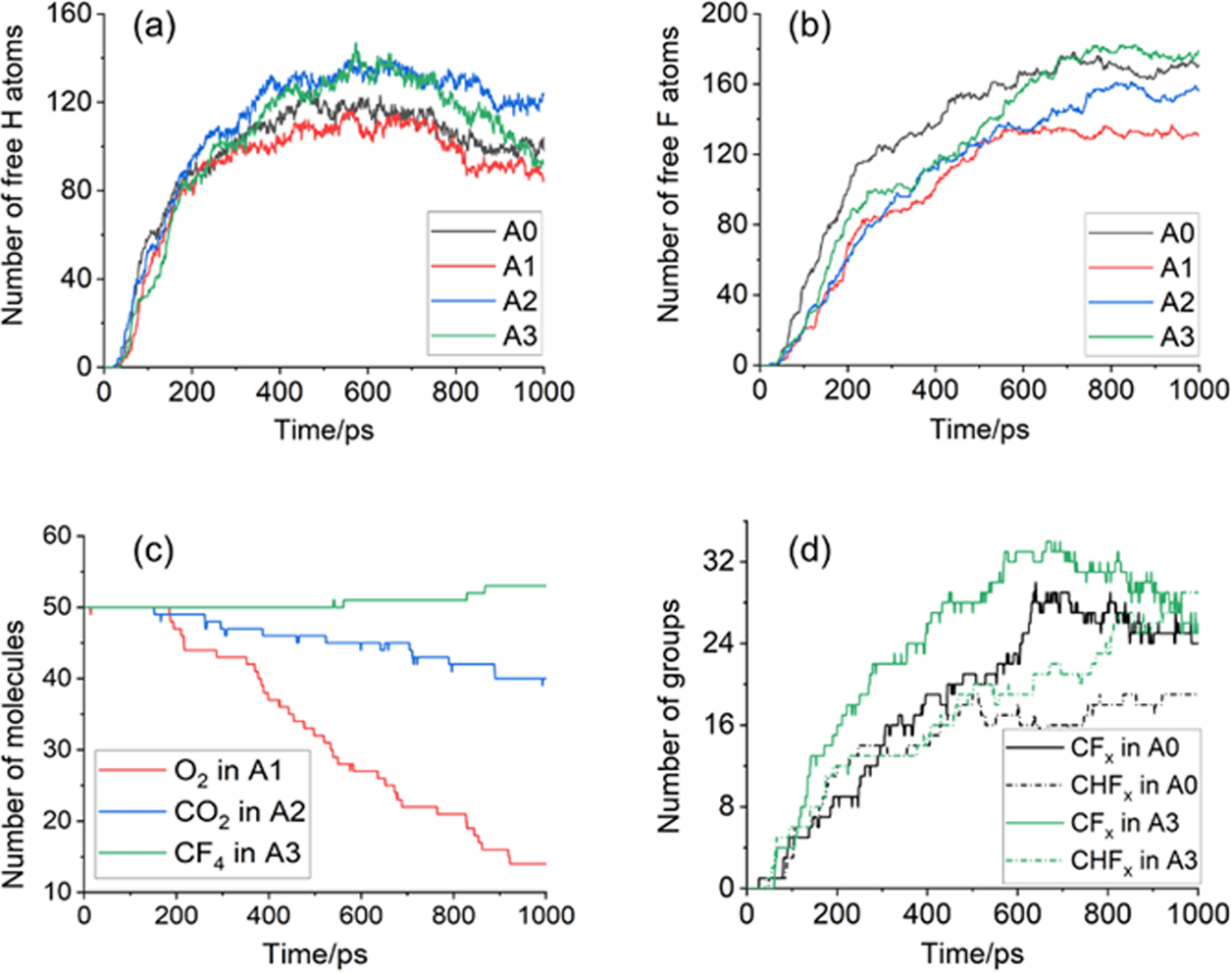
Comparison of main molecules (groups) contents in different systems with a number of (a) free H atoms; (b) free F atoms; (c) O_2_, CO_2_, and CF_4_ molecules; and (d) CF*_x_* and CHF*_x_* groups.

Next, we will further analyze the influence mechanism of different buffer gases on the amount of HFO solid precipitates based on the product curves of different buffer gas reaction systems. The change curves of reactants and main decomposition products of the A0–A3 system are shown in Figure 2. The comparison of product contents in different systems is shown in Figure 3. The typical reaction paths related to the following analysis are shown in Table 2.

**Table 2:** Typical Reaction Paths Related to the Formation and Consumption of C*_x_* Particles and the Formation of Other Key Products

ID	reaction path	ID	reaction path
(6)	C_3_F → C_3_ + F	(12)	O + C_2_ → CO + C
(7)	C_3_F + N → C_2_ + CNF	(13)	C + O_2_ → CO_2_
(8)	C_2_HF → C_2_ + HF	(14)	CF_3_ + O → COF_2_ + F
(9)	C_2_F → C_2_ + F	(15)	C_2_H + CF_3_ → CHF_3_ + C_2_
(10)	C_3_ → C_2_ + C	(16)	C_2_HF + CF_2_ → CHF_2_ + C_2_F
(11)	O_2_ + C_3_ → C_2_ + CO_2_		

Comparing Figure 2a,b, it can be seen that after a small amount of O_2_ is introduced into the HFO–N_2_ mixture, the free O atoms formed by the decomposition of O_2_ are easily combined with the free H atoms generated by the decomposition of HFO. During the 1000 ps simulation, the average number of H atoms in the A1 system was 88, which was 8% lower than that of the A0 system (96). Therefore, the presence of O_2_ inhibits the number of free H atoms in the system to a certain extent, thereby reducing the decomposition of HFO through the reaction path (4). Less decomposition of HFO molecules can inhibit the generation of C*_x_* particles from the source because the C-containing groups generated by the decomposition of HFO will be further decomposed under the influence of high temperature or other groups (such as reaction paths (6)–(10)) to generate C*_x_* particles. On the other hand, stable molecules such as CO, CO_2_, and COF_2_ are generated (reaction paths (11)–(14)) in the A1 system, which consumes the C*_x_* particles directly or indirectly. Under the joint action of the above two factors, the A1 system exhibits an excellent suppression effect of HFO solid precipitates. At the same time, less HFO decomposition can also improve the stability of the HFO gas mixture insulation performance to a certain extent.

It can be seen from Figure 3c that, as a stable oxidation product, the buffer gas CO_2_ has a lower reaction activity than O_2_ and so its decomposition amount during the reaction is less than O_2_. In addition, because a CO_2_ molecule can only provide one O atom (in this case, no new C particles are generated) for consuming C*_x_* particles or H atoms in the system, the inhibitory effect of CO_2_ on the decomposition amount of HFO and the promotion effect of CO_2_ on the consumption of C_x_ particles are not as obvious as that of O_2_.

From Figure 3d, it can be found that the presence of CF_4_ promotes the generation of CF*_x_* groups in the A3 system. The average content of CF*_x_* groups during the 1000 ps simulation increases by 30% compared to the A0 system. According to Table 1, the increase of the concentration of CF_3_ groups will promote the reverse reaction rate of the reaction path (3), which will reduce the amount of HFO decomposition. In addition, the average content of CHF*_x_* in 1000 ps is 21% higher than that of the A0 system. This is because the CF*_x_* particles present in the A3 system are more likely to take away the H or F atoms from the unstable C*_x_*H*_y_* or C*_x_*F*_y_* groups to form CHF_2_, CHF_3_, and C*_x_* particles (reaction paths (15)–(16)). Therefore, although the CF_4_ buffer gas suppresses the decomposition of HFO, it increases the generation of C*_x_* particles to a considerable degree. In short, the gas mixing scheme of the A3 system did not achieve the expected research purpose of suppressing the precipitation of solid products.

From the above analysis, it can be inferred that inhibiting the decomposition of HFO and promoting the consumption of C*_x_* particles all have a considerable impact on inhibiting the formation of the HFO solid precipitates. Therefore, in an ideal situation, the buffer gas needs to meet the two requirements at the same time to achieve the best suppression effect of the solid products. Among the several candidate buffer gases studied in this paper, only O_2_ satisfies these two requirements well and stands out in the screening process. In the following section, we will consider O_2_ as a potential buffer gas, and carry out further simulation and experimental research on the decomposition and insulation properties of the HFO–N_2_–O_2_ gas mixture.

### 2.2 Study on the Decomposition Characteristics of the HFO–N_2_–O_2_ Gas Mixture

#### 2.2.1 Influence of O_2_ Content on the Decomposition Characteristics of the HFO Gas Mixture

**Figure 4:**
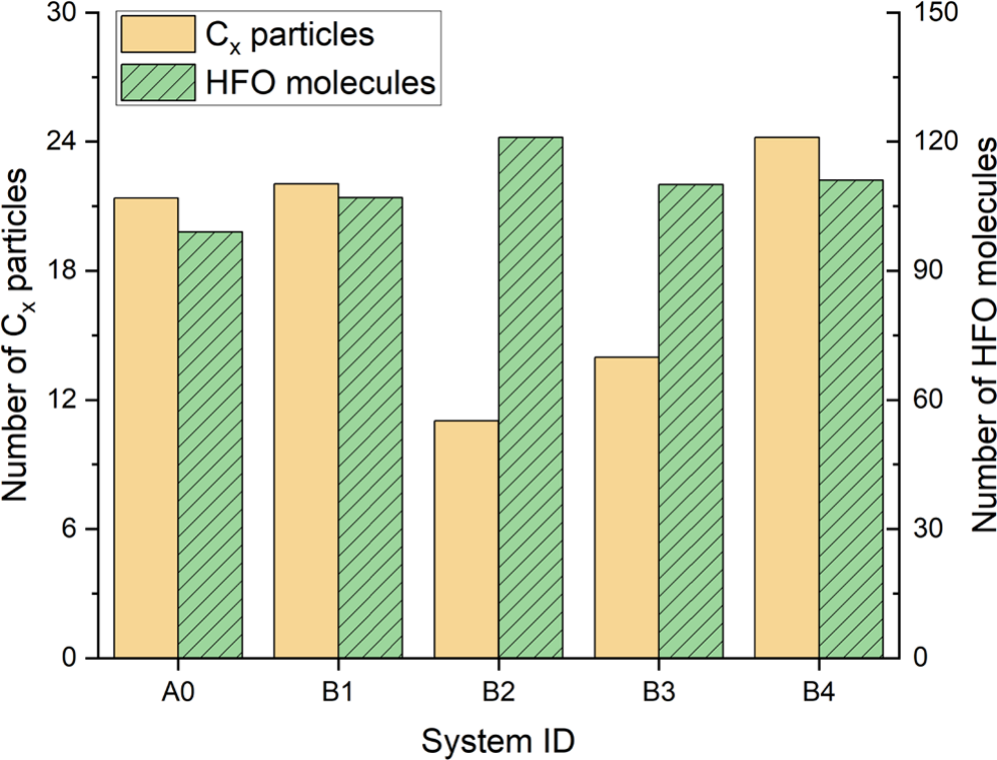
Number of C*_x_* particles and HFO molecules in the simulation system.

**Figure 5:**
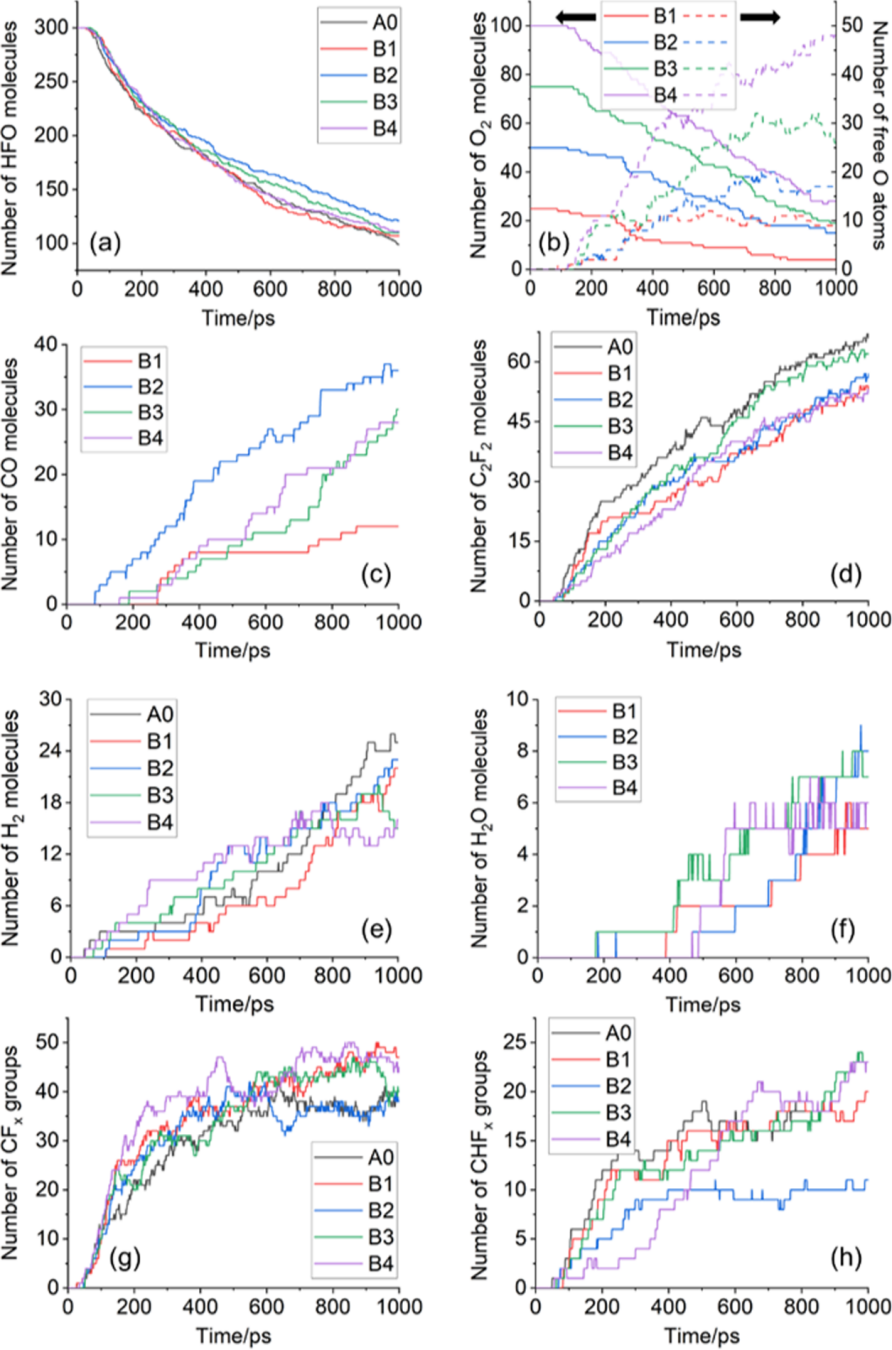
Change curves of the reactants and main decomposition products in the simulation systems with different oxygen contents: (a) a number of HFO molecules; (b) a number of O_2_ molecules; (c) a number of CO molecules; (d) a number of C_2_F_2_ molecules; (e) a number of H_2_ molecules; (f) a number of H_2_O molecules; (g) a number of CF*_x_* groups; and (h) a number of CHF*_x_* groups.

After performing 1000 ps simulation according to the settings of Section 4.1, the number of C*_x_* particles (average value during 900–1000 ps) and the number of remaining HFO molecules in systems with different O_2_ contents are shown in Figure 4. Figure 5 shows the change curves of the reactants and the main decomposition products in the simulation systems with different O_2_ contents.

It can be seen from Figure 4 that with the increase of the O_2_ content, the number of C*_x_* particles generated by the decomposition of HFO changes in a “V” shape. The O_2_ content in the B1 system is very small and so its inhibitory effect on HFO solid products is not obvious. When the O_2_ content reached 3.33% (B2 system), the amount of C*_x_* decreased significantly compared with that of the A0 system and the decrease rate reached 48%. Furthermore, as the content of O_2_ increases, the amount of C*_x_* gradually increased. Compared with the A0 system, the number of C*_x_* in the B4 system increased by 13%. On the other hand, under the influence of O_2_, the decomposition ratio of HFO in each system decreased to varying degrees. Compared with the decomposition ratio of HFO in the A0 system (67%), the decomposition ratio of HFO in the B2 system (60%) decreased significantly. However, the decomposition rate of HFO in B3 and B4 systems slightly increases than that of B2, reaching about 63%. The reason for this result can be explained from the perspective of the amount of O_2_ involved in the reaction. It can be seen from Figure 5b that in the system with higher O_2_ content, more free O atoms are generated. The analysis in Section 2.1 points out that free O atoms can reduce the probability of HFO decomposition via reaction path (4) by capturing free H atoms and can reduce the C*_x_* content by generating CO and CO_2_ products. However, when the content of free O atoms increases to a certain extent, the probability of O atoms directly attacking HFO molecules or C*_x_*H*_y_* and C*_x_*F*_y_* groups increases, thereby weakening the effect of O_2_ in inhibiting the HFO decomposition and C*_x_* particle generation. The related typical reaction paths are shown in Table 3.

**Table 3:** Typical Reaction Paths of O Atoms Directly Attacking HFO, C*_x_*F*_y_*, and C*_x_*H*_y_*

ID	reaction path
(17)	O + C_3_H_2_F_4_ → CF_3_ + C_2_HFO + H
(18)	O + C_3_F → C_2_ + COF
(19)	O + C_2_H → C_2_ + OH

According to the above analysis, the oxygen content in the HFO–N_2_–O_2_ mixture should be controlled at an appropriate level. The B2 system shows the best inhibition effects of solid products and the HFO decomposition.

In addition to the solid decomposition products, the presence of O_2_ will also affect the generation mechanism of other gas decomposition products such as CF_4_ and CHF_3_, which may have an important impact on the long-term insulation performance of the system.^22^ Therefore, it is necessary to analyze the decomposition characteristics of the HFO mixture under the influence of different O_2_ contents.

It can be seen from Figure 5c that with the increase of the O_2_ content in the system, the number of CO molecules produced by the reaction increases at first and then decreases. The B2 system produces the most CO molecules. The H_2_ content produced by the reaction is generally opposite to the change trend of the O_2_ content. The content of H_2_ generated in the B4 system is 36% lower than that of the A0 system. Trace amounts of H_2_O molecules are generated in simulation systems with different O_2_ contents but the O_2_ content has no obvious influence on the amount of H_2_O molecules generated. It can be seen from Figure 5g,h that the number of CF*_x_* and CHF*_x_* groups generated by decomposition in the B2 system is the smallest, which to a certain extent reflects that the B2 system has better long-term insulation properties.

#### 2.2.2 Influence of Temperature and Pressure on the Decomposition Characteristics of the HFO Gas Mixture

**Figure 6:**
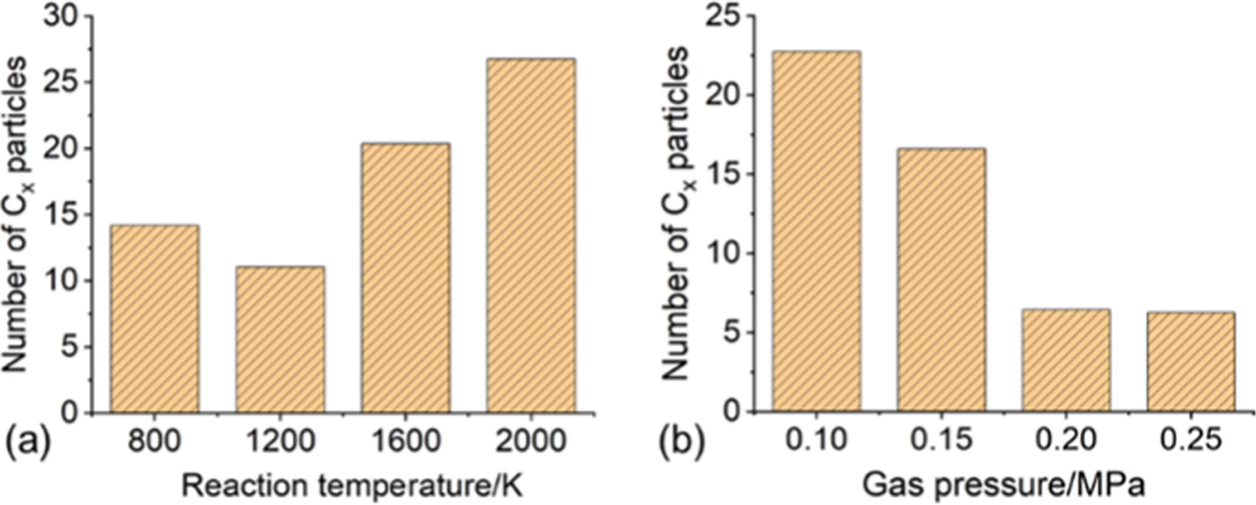
Number of C*_x_* particles generated by the decomposition of the HFO–N_2_–O_2_ gas mixture at different (a) reaction temperatures and (b) gas pressures.

The number of C*_x_* particles generated at different reaction temperatures and different gas pressure (average value during 900–1000 ps) is shown in Figure 6. The change curves of the reactants and main decomposition products in the simulation systems at different reaction temperatures and gas pressures are shown in Figures S1 and S2 (tables and figures whose label starts with “S” can be found in the Supporting Information).

First, we will analyze the effect of temperature on the decomposition characteristics of the HFO–N_2_–O_2_ gas mixture. It can be seen from Figure 6a that as the temperature increases, the number of C*_x_* particles generated in the system first decreases and then increases. At 1200 K, the number of C*_x_* particles generated by HFO decomposition is controlled at the lowest level. It can be seen from Figure S1a that the number of HFO molecules decomposed at 800 K is relatively small; however, the number of C*_x_* particles produced at this temperature is more than that at 1200 K. This is mainly because at 800 K, the number of free O atoms in the system is relatively small and the particle migration rate is relatively low. The combination of these two factors reduces the probability of O atoms encountering C*_x_* particles. Even if the encounter occurs, the probability that the particles have sufficient energy to overcome the reaction barrier and produce oxygen-containing products is small. As a result, the C*_x_* particles produced by the decomposition of HFO cannot be effectively consumed. At higher reaction temperatures, the proportion of HFO gas decomposition increases significantly. However, due to the relatively low proportion of O_2_ in the gas mixture, the difference between the C*_x_* generated by HFO decomposition and C*_x_* particles consumed by O atoms reaches the minimum at 1200 K. When the temperature continues to increase, even if O_2_ fully participates in the reaction, a lot of C*_x_* particles are still accumulated in the system. Therefore, it is speculated that higher discharge energy will lead to more solid precipitation. That is, the severity of the discharge fault is closely related to the amount of solid precipitation.

It can be seen from Figure S1 that the decomposition rate of HFO is greatly affected by the temperature. At higher temperatures, the decomposition amount of HFO is greater, which to a certain extent means that more solid products could be generated. At 800 and 1200 K, the HFO content curve basically shows a linear downward trend. However, when the temperature reaches above 1600 K, the HFO decomposition curve shows a clear logarithmic shape, that is, it decomposes rapidly at first and then the decomposition rate gradually decreases. Since the discharge intensity is directly related to temperature, optimizing the electrode structure or treating the electrode to reduce the discharge intensity is one of the possible methods to reduce the precipitation of HFO solid products.

On the other hand, O_2_ molecules start to decompose after 300 ps simulation at 800 K. This is mainly because the direct decomposition of the O=O double bond requires a large amount of energy to be absorbed. However, under the action of the free H and F atoms generated by the decomposition of HFO, the reaction barrier that needs to be overcome to break the O=O double bonds in O_2_ is greatly reduced. Therefore, when the H and F atoms in the system accumulate to a certain extent, the decomposition of O_2_ atoms can be effectively promoted.

At the reaction temperature of 800 K, no H_2_O molecules were generated in the system. At temperatures ranging from 1200 to 2000 K, the content of H_2_O generated in the system is basically at the same level. Compared with the content of other gas products, the amount of H_2_O generated is very small. So, the higher fault temperature will not have a significant impact on the moisture content of the HFO–N_2_–O_2_ gas mixture after a breakdown. The contents of C_2_F_2_, H_2_, CF*_x_*, and CHF*_x_* molecules (groups) increase significantly with the increase of the reaction temperature. The contents of C_2_F_2_ and H_2_ molecules showed a certain saturation tendency at the end of the 1000 ps simulation but still maintained a certain increasing rate. The contents of CF*_x_* and CHF*_x_* groups had reached saturation at about 400 ps. The contents of CF_4_ and CHF_3_ can be used to characterize the severity of discharge failures in the HFO–N_2_–O_2_ gas mixture insulation equipment.

Next, the influence of pressure on the decomposition characteristics of the HFO–N_2_–O_2_ gas mixture will be analyzed. It can be seen from Figure 6b that as the gas pressure increases, the number of C*_x_* particles produced by the reaction at the same temperature shows a decreasing trend. This is mainly because the higher gas pressure could increase the number of free H atoms, F atoms, and O atoms in the unit space, thus promoting the combination process of these particles with C*_x_* particles. The number of C*_x_* particles generated under 0.20 MPa pressure is only about one-fourth of that under 0.10 MPa. However, continuing to increase the pressure of the system has little effect on the suppression of the number of C*_x_* particles. This is because the increase in pressure will also increase the probability of H, F, and O attacking HFO molecules, thus promoting the decomposition of HFO. It can be seen from Figure S2a that compared with 0.20 MPa, the HFO decomposition amount at 0.25 MPa increased from 73 to 78%. More HFO decomposition will yield more C*_x_* particles. Under the influence of these two competitive factors, the number of C*_x_* particles approaches the minimum value at 0.20 MPa. The simulation results revealed that appropriately increasing the pressure of the HFO–N_2_–O_2_ gas mixture is a possible way to suppress the formation of solid products.

It can be seen from Figure S2 that the decomposition ratio of HFO and O_2_ increases slightly with the increase of gas pressure. Under different pressures, the decomposition rate of HFO in the initial stage is basically the same. It was not until about 50 ps that the curves showed relatively obvious differences. The analysis in the article shows that HFO is more likely to decompose when combined with free radicals such as H or F. Therefore, the increase in pressure mainly affects the decomposition rate of HFO by increasing the probability of HFO being attacked by other free radicals.

The formation of CO molecules is greatly affected by the gas pressure. The amount of CO molecules generated at 0.15 MPa is 50% higher than that at 0.10 MPa. However, when the gas pressure continues to increase, the amount of CO generated decreases on the basis of the amount at 0.15 MPa. Increasing the pressure of the gas mixture will significantly increase the content of H_2_O generated by decomposition. When the gas pressure reaches 0.25 MPa, the amount of H_2_O generated in the reaction system is about two times that under 0.10 MPa. Therefore, when considering the suppression of the solid product precipitation by increasing the gas pressure, additional attention needs to be paid to the influence of the generated H_2_O on the insulation strength of the gas–solid insulation system. When the system pressure is increased from 0.10 to 0.15 MPa, the contents of H_2_ and C_2_F_2_ will increase slightly; however, in the pressure range of 0.15–0.25 MPa, the pressure has little effect on the production of H_2_ and C_2_F_2_ molecules. The content of CF*_x_* groups is not significantly affected by air pressure. The content of CHF*_x_* groups increases as the pressure of the gas mixture increases and reaches saturation at 0.20 MPa.

#### 2.2.3 Formation Mechanism of the Main Products During the Diffusion Cooling Process

In this study, ReaxFF MD simulation was used to simulate the decomposition process of HFO gas molecules at high temperatures caused by the discharge fault. After 1000 ps ReaxFF MD simulation, in addition to stable products such as C_2_F_2_, CO, and H_2_, there are also many intermediate transition products such as H, F, CN, CF_2_, and CF_3_ with higher reactivity in the simulation system. The content of the main decomposition products in the simulation system at different reaction temperatures can be found in Figure S3. In the actual gas chamber, these intermediate products will diffuse into the lower temperature space around the discharge area and form various stable decomposition products through a relatively slow free radical recombination process.

Based on the stable products and free radicals obtained from MD simulations, the energy changes of various possible reaction paths are obtained by density functional theory (DFT) calculations. In the calculation, the Gaussian 09 quantum chemistry package and M062X/6-311+G(d,p) level were used.^24^ The calculated reaction paths and relative energy changes are shown in Table 4.

**Table 4:** Possible Reaction Paths and Relative Energy Changes (M06-2X/6-311+G(d,p))

ID	reaction path	standard reaction enthalpy (kJ/mol)
P1	CF_3_ + CF_3_ = C_2_F_6_	–405.48
P2	CF_2_ + CF_2_ = C_2_F_4_	–302.03
P3	CF_3_ + F = CF_4_	–532.47
P4	C_2_F_2_ + 2F = C_2_F_4_	–814.96
P5	C_2_F_4_ + 2F = C_2_F_6_	–810.97
P6	CF_3_ + CCF = CF_3_CCF	–531.04
P7	CF_3_ + CN = CF_3_CN	–480.48
P8	CO + O = CO_2_	–775.16
P9	H + OH = H_2_O	–485.76
P10	CF_3_ + H = CHF_3_	–442.44
P11	C_2_F_2_ + 2H = C_2_F_2_H_2_	–734.73

It can be seen from Table 4 that the reaction paths P1–P11 are all exothermic reactions. For this type of reaction, the more negative the standard reaction enthalpy, the greater the heat released by the reaction and, to a certain extent, the easier the reaction occurs.

Combining Figure S3 and Table 4, it can be seen that at the end of the 1000 ps reaction, the content of C_2_F_2_ and F accumulated in the system is large and so it is easy to generate C_2_F_4_ through path P4. On this basis, C_2_F_4_ can further generate C_2_F_6_ through path P5. At the same time, C_2_F_2_ can also combine with H atoms through path P12 to generate C_2_F_2_H_2_. The CO accumulated in the system can further react with free O atoms through path P9 to generate CO_2_. Taking into full consideration of the free radical content in the system and the energy change of the reaction path, it can be inferred that C_2_F_4_, C_2_F_6_, CO_2_, C_2_F_2_H_2_, CF_3_CCH, and CF_3_CCF are the main products generated in the diffusion cooling process. This result is basically consistent with the HFO decomposition products obtained by gas chromatography in the previous work of our research team.^22^ However, it does not reflect the formation of *cis*-C_3_H_2_F_4_ (the isomer of HFO1234Ze(E)), C_2_HF_3_, and CF_3_CHCF_2_.

### 2.3 DC Breakdown and the Solid Product Precipitation Characteristics of the HFO–N_2_–O_2_ Ternary Gas Mixture

#### 2.3.1 Solid Product Precipitation Characteristics

Using the experimental settings in Section 4.2, experiments on the breakdown characteristics of HFO–N_2_–O_2_ ternary gas mixtures under different pressures were carried out. Table 5 shows the mass measurement results of the solid decomposition products on the plate electrode before and after the breakdown test. The surface state of the ball electrode after the breakdown experiment is shown in Figure S4.

**Table 5:** Mass of Solid Decomposition Products on the Plate Electrode (mg)

	after 10 consecutive breakdowns		after 20 consecutive breakdowns	
	0.10 MPa	0.15 MPa	0.20 MPa	0.25 MPa
HFO–N_2_	1.1	0.8	0.9	0.8
HFO–N_2_–O_2_	0.4	0.4	0.3	0.4

It was found that the diameter of the area covered by the black solid products on the ball-plate electrode was about 1.38 cm. Most of the black products on the electrode surface can be easily removed. However, the black product within a diameter of about 3 mm (the area where the breakdown channel occurs) near the minimum distance of the ball-plate electrode is tightly attached and cannot be directly removed, as shown in Figure S4b. This is because a large amount of energy is released when the gas gap breakdown occurs. The high temperature near the breakdown channel causes the black product to adhere tightly to the surface of the ball electrode. In addition, after a single breakdown test of the HFO–N_2_–O_2_ gas mixture, a large area of black solid precipitation was also observed. Combining the experimental phenomena with the electric field simulation results, it can be found that the area where the black solids are distributed is basically the same as the area where the electric field is severely distorted.

Based on the above analysis, it is speculated that the black solid decomposition products are mainly generated by the corona discharge between the ball plate electrodes before the air gap breakdown. However, the reliability of this speculation still cannot be fully proved according to the current experimental results and so further research is needed.

Furthermore, the area of the solid decomposition product adhesion area on the electrode surface in HFO–N_2_ and HFO–N_2_–O_2_ environments under the same pressure was compared. The results show that the diameter of the black solid distribution area did not change significantly, but the color near the edge of the black area was slightly lighter after adding O_2_. It can be seen from Table 5 that under the same pressure, the mass of solids decomposed in the HFO–N_2_–O_2_ gas mixture is significantly reduced compared with that in the HFO–N_2_ gas mixture. In addition, with the increase of gas pressure, the solid content generated under the same gas shows a downward trend. The above two experimental phenomena are consistent with those observed in ReaxFF MD simulation.

#### 2.3.2 DC Breakdown Characteristics

**Figure 7:**
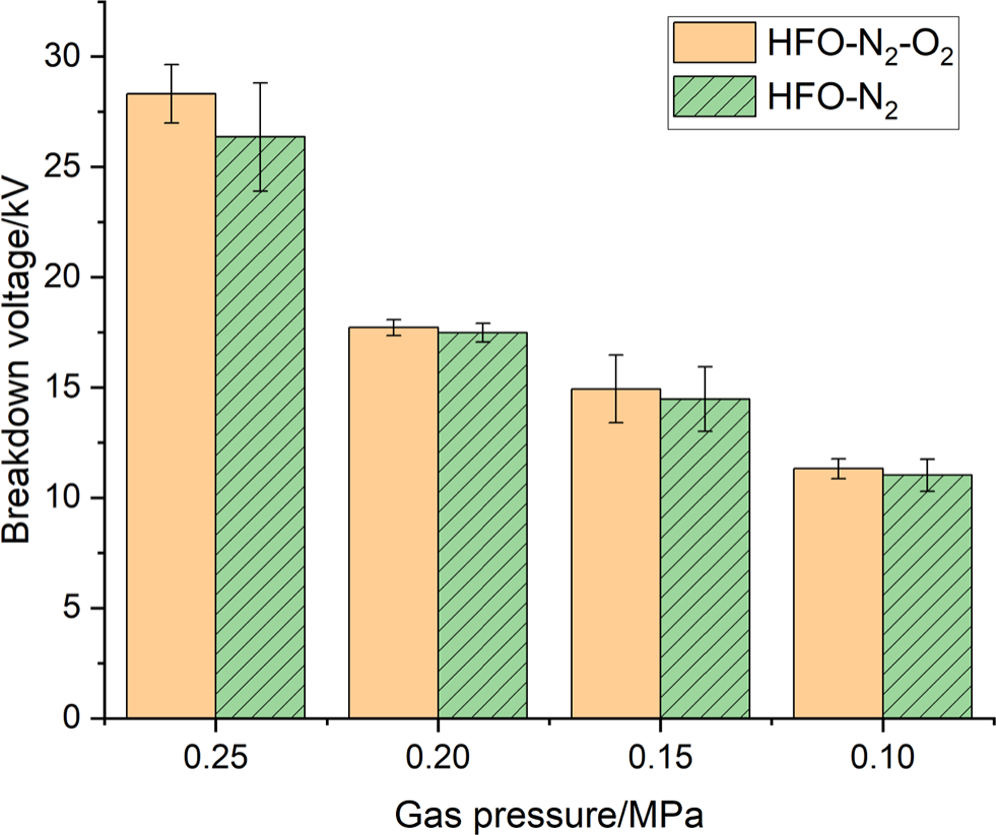
Breakdown voltage of the HFO–N_2_–O_2_ gas mixture at different gas pressures.

To visually compare the effect of O_2_ on the breakdown voltage of the gas mixture, we calculated the average value and the mean square error of the 2nd–6th breakdown voltage under each experimental setting (the reason for ignoring the first breakdown voltage will be explained later); the result is shown in Figure 7. It can be seen from Figure 7 that after adding O_2_ to the HFO–N_2_ gas mixture, the breakdown voltage at the same pressure increases slightly. This is because the electronegativity of oxygen is stronger than that of nitrogen and so O_2_ is better than N_2_ in absorbing free electrons, which makes collision ionization relatively weaker during discharge.

**Figure 8:**
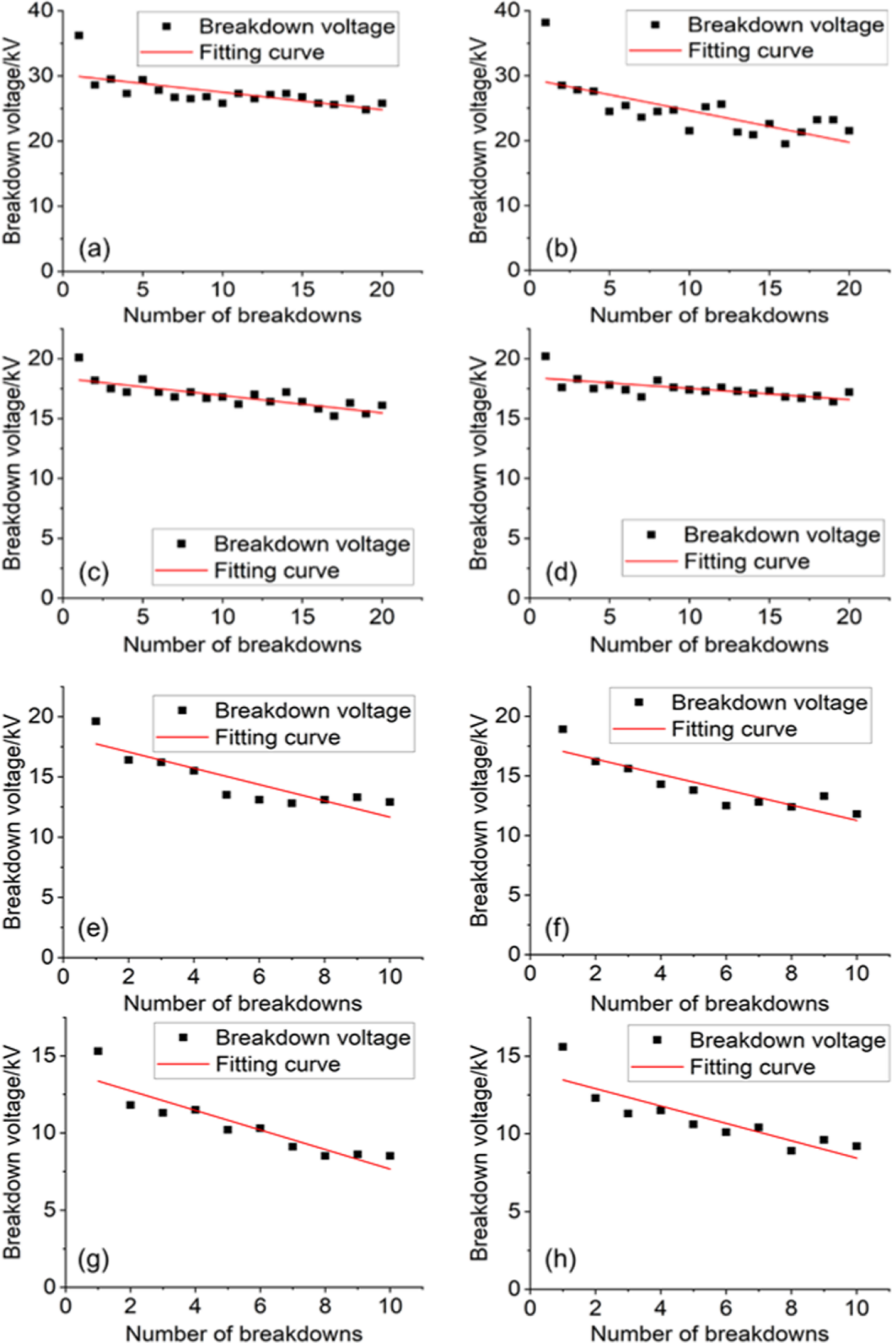
Variation of the breakdown voltage with breakdown times: (a) 0.25 MPa HFO–N_2_–O_2_; (b) 0.25 MPa HFO–N_2_; (c) 0.20 MPa HFO–N_2_–O_2_; (d) 0.20 MPa HFO–N_2_; (e) 0.15 MPa HFO–N_2_–O_2_; (f) 0.15 MPa HFO–N_2_; (g) 0.10 MPa HFO–N_2_–O_2_; and (h) 0.10 MPa HFO–N_2_.

The self-recovery characteristics and breakdown voltage dispersion degree of the HFO gas mixture are important indicators for evaluating gas insulation performance. The change of breakdown voltage with the number of breakdowns is shown in Figure 8. Comparing the breakdown voltage of HFO–N_2_ and HFO–N_2_–O_2_ gas mixtures at the same pressure, it can be found that the addition of O_2_ slows down the drop rate of the breakdown voltage to a certain extent. At 0.25 MPa pressure, for example, the fitting curve slope of the HFO–N_2_–O_2_ gas breakdown voltage is −0.2685, which is 45% lower than that of the HFO–N_2_ gas mixture (−0.4887). In addition, the presence of O_2_ also reduces the dispersion degree of breakdown voltage, and this phenomenon is more obvious under relatively high gas pressure.

In addition, it can be observed from Figure 8 that regardless of the presence or absence of O_2_, there is a relatively large voltage drop (in some cases, the drop can reach 15–20%) between the first breakdown voltage and the second breakdown voltage of the HFO mixture. After the second breakdown, although the subsequent breakdown voltage will gradually decrease with the increase in the number of breakdowns, the decrease is far less than the drop between the first and second breakdown voltages. Based on experimental phenomena, it is speculated that the black decomposition products attached to the electrode are the main reason for the initial voltage drop. When solid products are attached to the electrode surface, the surface roughness increases significantly and there are many protrusions on the microstructure. Therefore, the electric field on the electrode surface will be greatly distorted, which in turn is more likely to cause partial discharge and even breakdown.

It is noticed that the addition of O_2_ has almost no obvious effect on the initial voltage drop. This may be because the electrode surface is in good condition before the first breakdown and so the corona discharge intensity is relatively low, and the corresponding reaction zone temperature is also lower. At this time, O_2_ cannot fully participate in the reaction to consume C*_x_* particles (this phenomenon is consistent with the simulation result of the lower reaction temperature). In the subsequent breakdown process, however, the solid precipitates caused more serious corona discharge; at this time, O_2_ inhibition of the solid precipitate was more obvious. This can explain why O_2_ improves the degree of subsequent breakdown voltage drop.

Based on the above analysis, it can be concluded that adding a small amount of O_2_ to the HFO–N_2_ gas mixture can effectively inhibit the generation of solid decomposition products and will not negatively affect the gas insulation characteristics. Therefore, O_2_ is a promising buffer gas for suppressing HFO solid products, and it is worth continuing to conduct more in-depth research.

### 2.4 Discussion

#### 2.4.1 Universality of Research Methods

The discussion in the previous section shows that the ReaxFF MD simulation and experimental results have satisfactory consistency in the characterization of solid decomposition products and the selection of HFO gas mixing schemes. However, the novel analysis method proposed in this paper is not limited to HFO. With the support of a reliable ReaxFF force field, the application of ReaxFF MD simulation has great expansibility.

The ReaxFF force field used in this study comes from the literature research, mainly including C, H, F, O, and N elements and has been widely used in the study of the decomposition characteristics of gases containing these elements. The above analysis can further illustrate that the gas mixing scheme screening method based on the ReaxFF MD simulation proposed in this paper can also provide effective technical support for solving the problem of solid precipitation suppression of other environmentally friendly insulating gas in the C/F/O/N system (such as C_4_F_7_N, C_5_F_10_O, etc.).

#### 2.4.2 Problems that Require Further Study

In the decomposition experiment of the insulating gas mixture C_4_F_7_N–N_2_–O_2_, which has similar properties to the HFO–N_2_–O_2_ gas mixture, the COF_2_ molecule is detected. Relevant studies have shown that COF_2_ has strong biological toxicity and is highly corrosive.^25^ Therefore, the production of this substance will cause damage to the health of operation and maintenance personnel and the safe operation of equipment. Under the simulation setting of this article (3.33% oxygen content), a few COF_2_ molecules are generated at 800–2000 K. However, considering that the temperature of the fault discharge area in the gas-insulated equipment may be much higher than the temperature used in the simulation, it is necessary to carry out further experimental research on the content of harmful substances such as COF_2_ in the HFO–N_2_–O_2_ gas mixture discharge decomposition products.

According to the experimental research carried out in this article, it is found that although the addition of O_2_ to HFO–N_2_ can inhibit the formation of solid products to a large extent, the problem of initial pressure drop remains unresolved. The decrease of the gas insulation strength after the initial breakdown is a huge test for equipment insulation so further research is urgently needed. On the other hand, to prove that O_2_ will not negatively affect the insulation properties of the HFO gas mixture, in this paper, we carried out an experimental study on the air gap breakdown characteristics in the slightly uneven electric field under a negative DC voltage. However, to fully demonstrate the application potential of O_2_ as a buffer gas for suppressing the solid products, further research is needed under different voltage conditions (such as AC voltage, impulse voltage, and AC/DC superimposed impulse voltage) and different electric field uniformities.

## 3 Conclusions

In this paper, simulations and experiments are performed to study the inhibitory effect of the buffer gas on the HFO solid decomposition products. The main conclusions obtained from this study are as follows:

(1) The inhibitory effect of O_2_ on the formation of HFO solid precipitates is better than that of CO_2_ and CF_4_. With the increase of the O_2_ content in the HFO–N_2_–O_2_ gas mixture, the content of solid precipitates first decreases and then increases and reaches the minimum when O_2_ accounts for 3.33% of the gas mixture, in which case the precipitate content decreased by 48% compared with that in the HFO–N_2_ gas mixture. In addition, the content of solid precipitates is proportional to the reaction temperature and inversely proportional to the gas pressure.
(2) Experimental research shows that compared with HFO–N_2_ (20/80%), the DC breakdown voltage of the HFO–N_2_–O_2_ (20/76.67/3.33%) gas mixture increases slightly and the breakdown voltage dispersion degree is reduced. In addition, the continuous breakdown voltage drop rate of HFO–N_2_–O_2_ is also reduced to a certain extent. After the same number of breakdown tests, the mass of the solid products attached to the electrode is significantly reduced under the influence of O_2_ and the reduction percentage could reach more than 50%. Therefore, O_2_ as a buffer gas has a prominent application prospect in achieving the suppression of HFO solid precipitates.
(3) A buffer gas screening method for the suppression of the HFO solid decomposition products based on ReaxFF MD simulation is proposed. The cross-validation of simulation and experimental results supports the validity of the proposed research method. Based on the expandability of ReaxFF simulation, this method can be applied to solve the similar problem of environmentally friendly insulating gases in the C/F/O/N system such as C_4_F_7_N and C_5_F_10_O.

## 4 Methods

### 4.1 Simulation Methods

The ReaxFF force field used in this study contains C, H, O, N, and F elements,^26^ and has been applied to the study of the pyrolysis characteristics of HFO gas.^13^ To avoid the drastic change of system energy in the initial stage of simulation, the molecular structures of HFO, N_2_, and candidate buffer gases were optimized under the selected force field. Then the builder module integrated into AMS software is used to establish the periodic simulation model. The total number of molecules and gas pressure in the simulation system is set to 1500 and 0.1 MPa, respectively. Given the number of gas molecules, the gas pressure and temperature (298 K), the side length of the simulation cubic box should be 395.21 Å according to the ideal gas law. Based on the research results on the HFO gas insulation characteristics in the literature,^13,27^ the HFO–N_2_ (20/80%) gas mixture system with prominent application potential is selected as the benchmark in this research, identifying as A0. On this basis, keeping the content of HFO unchanged and then replacing 50 N_2_ molecules with O_2_, CO_2_, or CF_4_ molecules, respectively, to form three HFO ternary gas mixture systems, identifying as A1–A3. The detailed parameters of each simulation system are shown in Table S1.

The MD simulations in this study are all carried out under canonical ensemble (NVT). The temperature control method is Berendsen, the damping constant is 100 fs, and the simulation time step is 0.25 fs. First, 10 ps MD simulation is performed at a temperature of 298 K to make the system reach an equilibrium state. Then, the temperature of the simulation system is increased at a rate of 0.1 K/fs to 1200 K,^13^ and this temperature is maintained until the end of the simulation. A total of 1000 ps simulation was performed.

Using the same modeling method as before, 25, 50, 75, and 100 N_2_ molecules were replaced with O_2_ molecules, respectively, to establish four simulation systems with different oxygen contents, denoted B1–B4. The parameters of the simulation systems are shown in Table S2. Based on the gas mixing ratio of the B2 system model, four simulation models with 0.10, 0.15, 0.20, and 0.25 MPa gas pressures were established, denoted C1–C4. The parameter settings of the simulation system are shown in Table S3.

For the simulation systems in Tables S2 and S3, after performing 10 ps NVT relaxation at 298 K, the temperature of the simulation system is increased to 1200 K at a rate of 0.1 K/fs and maintained until the end of the 1000 ps simulation. To explore the effect of reaction temperatures on the decomposition characteristics of the HFO–N_2_–O_2_ gas mixture, a series of ReaxFF MD simulations were carried out in the B2 system model. After 10 ps NVT relaxation at 298 K, the temperatures of the simulation system were increased to 800, 1200, 1600, and 2000 K, respectively, at a rate of 0.1 K/fs and kept until the end of the 1000 ps simulation.

### 4.2 Experimental Methods

**Figure 9:**
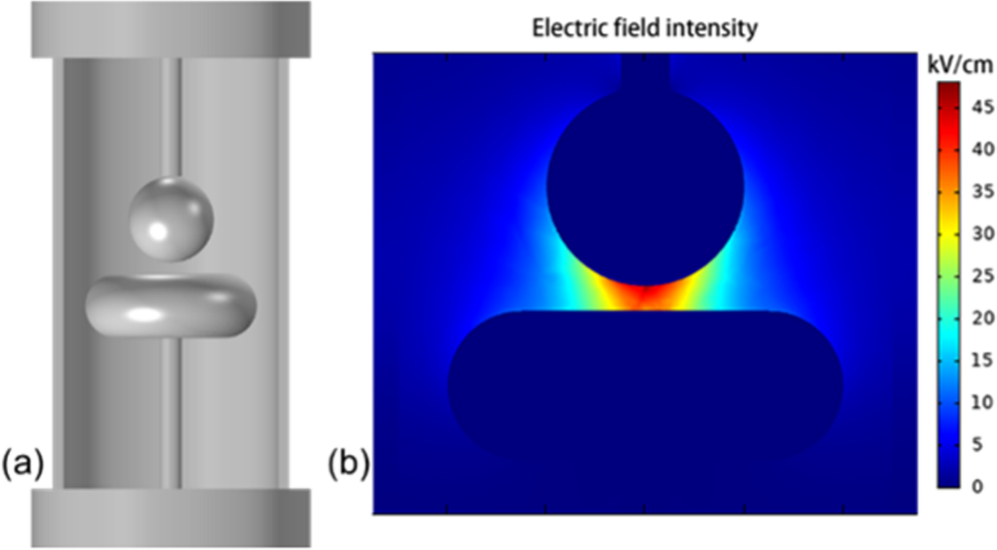
Related information of the gas insulation performance test platform: (a) structural diagram and (b) electric field simulation results.

In this study, the DC voltage is provided by the HYAC/DC-300 AC/DC power source. The breakdown test for the HFO–N_2_–O_2_ gas mixture is carried out in the gas insulation performance test platform as shown in Figure 9a. The radius of the ball electrode used in the experiment is 40 mm and the distance between electrodes is 5 mm. COMSOL Multiphysics software is used to calculate the electric field distribution of the ball-plate electrode model in Figure 9a. The electric field calculation result is shown in Figure 9b, where the plate electrode is used as the ground electrode. According to the simulation results, the maximum field strength and the average field strength of the electric field are obtained. The calculated electric field unevenness coefficient *f* = 1.12, which satisfies the definition of slightly uneven field (*f* < 2).

Before the test, the gas chamber was evacuated to −0.1000 MPa and filled with N_2_ (purity: 99.999%), and then evacuated again. Next, according to the proportion of each gas in the gas mixture (3.33/20/76.67%), O_2_ (purity 99.999%), HFO (purity 99.5%), and N_2_ are injected into the gas chamber successively. After the inflation is completed, the experimental cavity is left to stand for 12 h to fully mix the gas. After ensuring that the cavity is well sealed, subsequent operations were carried out.

In the experiment, a DC ramp voltage at an increased rate of 0.5 kV/s was applied until breakdown occurred to obtain the breakdown voltage of the HFO–N_2_–O_2_ gas mixture under a negative DC voltage. Through preliminary experiments, it was found that when the gas pressure is relatively low, the corona discharge phenomenon of the ball electrode after multiple breakdowns is relatively strong, which makes it difficult to obtain the breakdown voltage. Therefore, for the HFO–N_2_–O_2_ gas mixture with pressures of 0.1 and 0.15 MPa, 10 consecutive breakdown tests were performed. For the gas mixture at pressures of 0.2 and 0.25 MPa, 20 breakdown tests were carried out. In the continuous breakdown test, a breakdown is performed every 3 min. To quantitatively compare the influence of O_2_ on the generation of HFO solid decomposition products, a thin copper sheet (with a thickness of 0.5 mm) was placed on the plate electrode. The conductive glue was used to ensure reliable conduction between the copper sheet and the plate electrode. To avoid the residual conductive glue affecting the weighing result of solid precipitates, absolute ethanol was used to wipe off the conductive glue before weighing. The analytical balance ME104/02 (measurement accuracy 0.0001 g) was used to measure the weight change of the copper sheet before and after the breakdown test.

## Supporting Information Available

ReaxFF simulation results for the HFO–N_2_–O_2_ system; the surface state of the ball electrode after continuous breakdown test; and detailed parameters of the simulation systems (PDF)
