# Supplementary material for: Inhibition Effect of Solid Products and DC Breakdown Characteristics of the HFO1234Ze(E)–N2–O2 Ternary Gas Mixture
Source: ACS Omega. 2021 Aug 31;6(36):23281–92. doi: 10.1021/acsomega.1c03020 (PMC8444307; doi:10.1021/acsomega.1c03020)
Supplement: Supplementary file 1 — ao1c03020_si_001.pdf [file ao1c03020_si_001.pdf]

# Inhibition Effect of Solid Products and DC Breakdown Characteristics of the HFO1234Ze(E)-$N_2$-$O_2$ Ternary Gas Mixture

Heng Liu[1], Qingmin Li[1*], Jingrui Wang[1], Yuheng Jiang[2], A. Manu Haddad[3]

1 State Key Laboratory of Alternate Electrical Power System with Renewable Energy Sources, North China Electric Power University, Beijing 102206, China

2 School of Electrical and Electronic Engineering, North China Electric Power University, Beijing 102206, China

3 Advanced High Voltage Engineering Research Centre, Cardiff University, Cardiff, Wales CF24 3AA, United Kingdom

# Supporting Information

## 1. ReaxFF simulation results for HFO-$N_2$-$O_2$ system

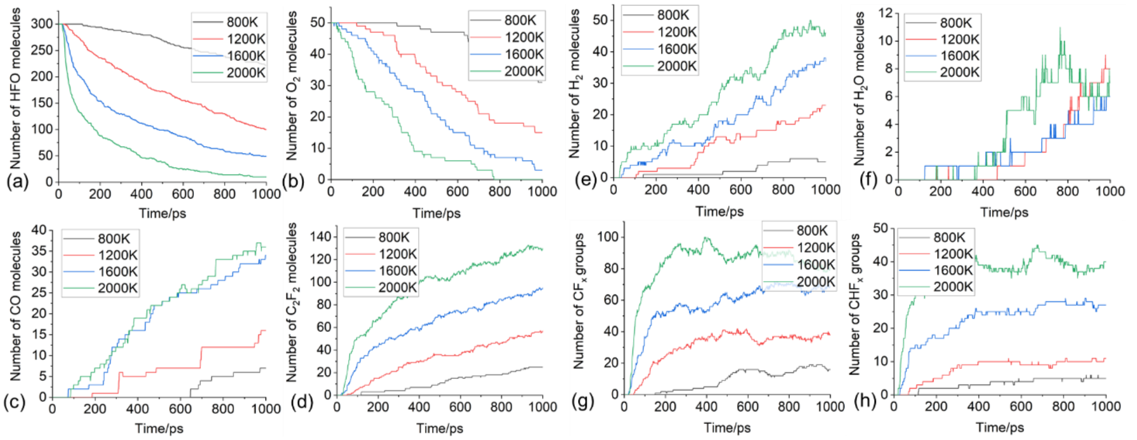


**Figure S1.** The content of reactants and main decomposition products in HFO-$N_2$-$O_2$ system at different reaction temperatures: (a) number of HFO molecules; (b) number of $O_2$ molecules; (c) number of CO molecules; (d) number of $C_2F_2$ molecules; (e) number of $H_2$ molecules; (f) number of $H_2O$ molecules; (g) number of $CF_x$ groups; (h) number of $CHF_x$ groups.

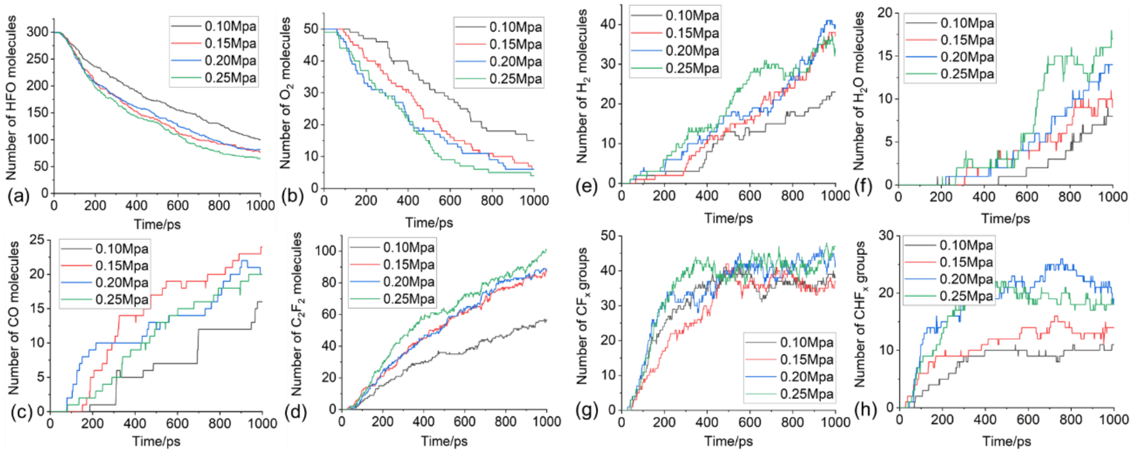


**Figure S2.** The content of reactants and main decomposition products in HFO-$N_2$-$O_2$ system at different pressures: (a) number of HFO molecules; (b) number of $O_2$ molecules; (c) number of CO molecules; (d) number of $C_2F_2$ molecules; (e) number of $H_2$ molecules; (f) number of $H_2O$ molecules; (g) number of $CF_x$ groups; (h) number of $CHF_x$ groups.

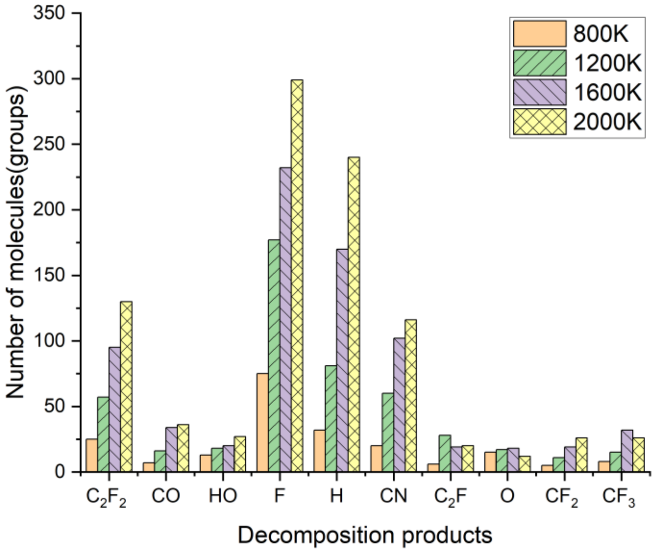


**Figure S3.** The content of main radicals of HFO-$N_2$-$O_2$ simulation system at different temperatures.

## 2. Experimental results

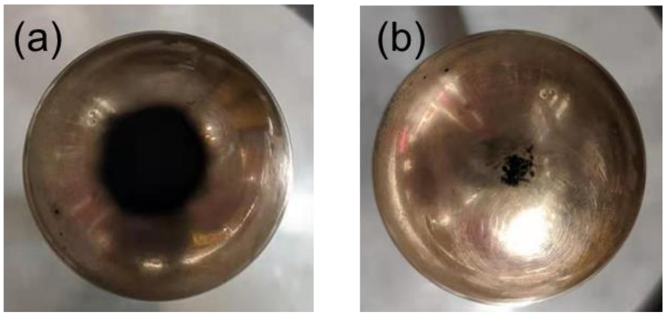


**Figure S4.** The surface state of the ball electrode after continuous breakdown tests: (a) before wiping; (b) after wiping.

## 3. The detailed parameters of the simulation system

**Table S1.** Simulation system parameters of HFO ternary mixture with different buffer gases

| System ID | Pressure/MPa | Number of molecules | Side length/Å |
|---|---|---|---|
| A0 | 0.10 | 300HFO/1200$N_2$ | 395.21 |
| A1 | 0.10 | 300HFO/1150$N_2$/50$O_2$ | 395.21 |
| A2 | 0.10 | 300HFO/1150$N_2$/50$CO_2$ | 395.21 |
| A3 | 0.10 | 300HFO/1150$N_2$/50$CF_4$ | 395.21 |

**Table S2.** Simulation system parameters of HFO-$N_2$-$O_2$ gas mixture with different $O_2$ content

| System ID | Pressure/ MPa | Number of molecules | Side length/ Å |
|---|---|---|---|
| A0 | 0.10 | 300HFO/1200$N_2$ | 395.21 |
| B1 | 0.10 | 300HFO/1175$N_2$/25$O_2$ | 395.21 |
| B2 | 0.10 | 300HFO/1150$N_2$/50$O_2$ | 395.21 |

| | | | |
|---|---|---|---|
| B3 | 0.10 | $300HFO/1125N_2/75O_2$ | 395.21 |
| B4 | 0.10 | $300HFO/1100N_2/100O_2$ | 395.21 |

**Table S3.** Simulation system parameters of HFO-$N_2$-$O_2$ gas mixture with different gas pressures

| System ID | Gas pressure/ MPa | Number of molecules | Side length/ Å |
|---|---|---|---|
| C1 | 0.10 | $300HFO/1150N_2/50O_2$ | 395.21 |
| C2 | 0.15 | $300HFO/1150N_2/50O_2$ | 345.25 |
| C3 | 0.20 | $300HFO/1150N_2/50O_2$ | 313.68 |
| C4 | 0.25 | $300HFO/1150N_2/50O_2$ | 291.20 |
